# Phylogenetic placement of the Pacific Northwest subterranean endemic diving beetle *Stygoporus oregonensis* Larson & LaBonte (Dytiscidae, Hydroporinae)

**DOI:** 10.3897/zookeys.632.9866

**Published:** 2016-11-16

**Authors:** Kojun Kanda, R. Antonio Gomez, Richard Van Driesche, Kelly B. Miller, David R. Maddison

**Affiliations:** 1 Department of Integrative Biology, Oregon State University, Corvallis, Oregon, United States of America; 2 Department of Biology, University of New Mexico, Albuquerque, New Mexico, United States of America

**Keywords:** Stygobiont, aquatic Coleoptera, Hydroporini, aquifer, Siettitiina, Nearctic, Oregon

## Abstract

*Stygoporus oregonensis* Larson & LaBonte is a little-known subterranean diving beetle, which, until recently, had not been collected since the type series was taken from a shallow well in western Oregon, USA, in 1984. Here we report the discovery of additional specimens collected from a nearby well in the Willamette Valley. Sequence data from four mitochondrial genes, *wingless*, and histone III place *Stygoporus* Larson & LaBonte in the predominantly Mediterranean subtribe Siettitiina of the Hydroporini. Morphological support for these results is discussed, and details of the collecting circumstances of the new specimens are presented. We argue that the biogeographic patterns of Nearctic Siettitiina highlight the likelihood of additional undiscovered subterranean dytiscids in North America.

## Introduction

In the spring of 1984 an unusual, pale, blind diving beetle was found in a bathtub in a private residence near the town of Dallas, Oregon, USA. The bathtub received water directly from a shallow well that was drawing from the Willamette Lowland aquifer system in the central Willamette Valley. The residents sent the specimen to an entomology extension specialist, Dr. J. Capizzi at Oregon State University, who recognized the beetle as distinct and suggested to the residents that they collect more specimens (Larson and LaBonte 1994). An additional eight specimens were found, and shortly thereafter, the residents treated the well with chlorine. No additional specimens were collected at the type locality following the well’s chlorine treatment (Larson and LaBonte 1994). The species was described and given the name *Stygoporus oregonensis* Larson & LaBonte in honor of its subterranean predilections and the state from which it was thus far known (Larson and LaBonte 1994). In the more than 30 years since the type series was collected, no additional specimens of *Stygoporus oregonensis* have been reported prior to the present study.

**Figure 1.**
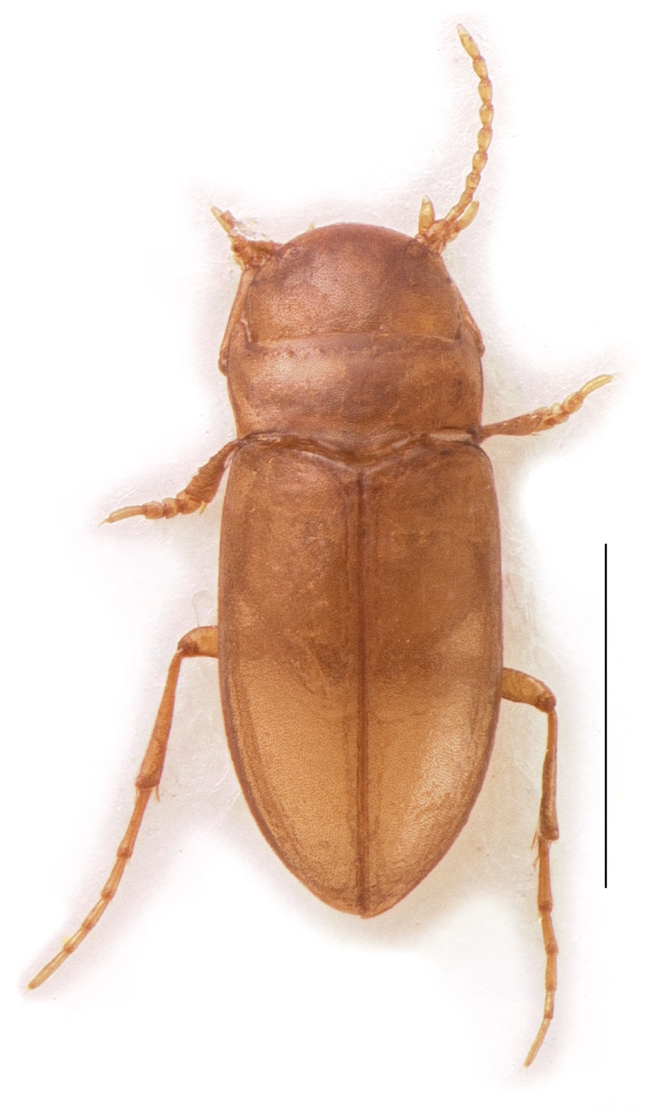
Dorsal habitus of female *Stygoporus oregonensis*. Scale bar = 1 mm.

*Stygoporus oregonensis* is a small-bodied diving beetle with pale, mostly yellow cuticle, long elytral marginal setae, fused elytra, minute flight wings, and without eyes (Larson and LaBonte 1994; Fig. 1). These morphological features are commonly observed in various, often widely unrelated subterranean lineages and are considered to typify stygobitic Dytiscidae from around the world (Leys and Watts 2008; Leys et al. 2003; Miller et al. 2013; Spangler and Decu 1998; Watts and Humphreys 2009). An additional morphological feature common among stygobitic dytiscids is a discontinuous body outline, contrasted with the more streamlined habitus of many diving beetles.

Inferring the phylogenetic placement of stygobitic species is crucial for shedding light on their origins and developing a framework for studying adaptation and other responses to subterranean environments. Addressing the mechanisms responsible for the unusual though oft-repeated appearance of the stygobitic fauna and their often unexpected distributions is an active field (Juan et al. 2010). Cave faunas are some of the most visually striking examples of convergence, and several recent studies of stygobitic and troglobitic life have used the character-rich information present in molecular sequence data to help place these morphologically similar species into phylogenetic hypotheses (Faille et al. 2010; Gómez et al. 2016; Leys et al. 2003; Miller et al. 2013; Ribera et al. 2010; Toussaint et al. 2015; Wiens et al. 2003).

In the United States, three aquatic beetle families are known to include stygobitic species: Dryopidae (*Stygoparnus comalensis* Barr & Spangler, 1992), Elmidae (*Typhloelmis* Barr, 2015: 3 species), and Dytiscidae (*Ereboporus naturaconservatus* Miller, Gibson & Alarie, 2009, *Haideoporus texanus* Young & Longley, 1976, *Psychopomporus felipi* Jean, Telles & Miller, 2012, *Comaldessus stygius* Spangler & Barr, 1995, and *Stygoporus oregonensis* Larson & LaBonte, 1994). Apart from *Stygoporus oregonensis*, all US stygobitic beetles are only known to occur in the Edwards-Trinity aquifer system in central Texas.

Whereas the relationships of *Stygoparnus* Barr and Spangler and *Typhloelmis* to other members of their respective families have yet to be explored with phylogenetic methods, the placement of three of the four described Texas stygobitic dytiscids within the very diverse subfamily Hydroporinae was recently inferred using molecular sequences (Miller et al. 2013). Miller et al. (2013) did not include *Comaldessus stygius* in their analyses because it possesses several morphological synapomorphies that unambiguously place it within Bidessini. The other Texas stygobites were placed in two clades, the *Graptodytes* group (*Ereboporus naturaconservatus* and *Psychopomporus felipi*) and the *Hydroporus* group (*Haideoporus texanus*). Both of these generic groups are traditionally classified within the large, heterogeneous tribe Hydroporini *sensu lato*, which has been shown to be polyphyletic by several authors (Miller et al. 2006; Ribera et al. 2002; Ribera et al. 2008). Recently, Miller and Bergsten (2014) formalized the subgroups of Hydroporini *s. l.* establishing the subtribe Siettitiina for *Graptodytes* group and Hydroporina for *Hydroporus* group; they also provisionally placed *Stygoporus oregonensis* in Hydroporina.

In their paper describing *Stygoporus oregonensis*, Larson and LaBonte (1994) hypothesized that *Stygoporus* is related to the Nearctic genus *Sanfilippodytes* Franciscolo (also placed in Hydroporina by Miller and Bergsten (2014)) based on similarly large metatrochanters, apically produced metaventral processes, and *Sanfilippodytes* exhibiting character states that “form a good base from which a truly subterranean beetle could evolve” (Larson and LaBonte 1994). In addition, several *Sanfilippodytes* species are known from a variety of habitats including acidic pools (Post 2010), interstitial spaces along margins of springs and creeks, within sand-clay or gravel substrate of cold springs, limnocrene pools, under beach debris or cover along the margins of alpine lakes, under mosses in springs and seeps, and caves (Larson et al. 2000), which may be steps along the way to colonization of subterranean aquifers by the ancestor of *Stygoporus oregonensis*. However the relationship between these genera has yet to be tested.

In this paper, we report additional specimens of *Stygoporus oregonensis* from a separate well, also in the central Willamette Valley, Oregon. These specimens yielded DNA, from which we amplified six genes used in Miller et al.’s (2013) phylogeny of Hydroporinae. We incorporate our new sequences with data from Miller et al. (2013) to infer the phylogenetic placement of *Stygoporus oregonensis* and discuss morphological aspects of *Stygoporus oregonensis* in light of these results.

## Methods

### Discovery of *Stygoporus oregonensis* specimens

**Figure 2.**
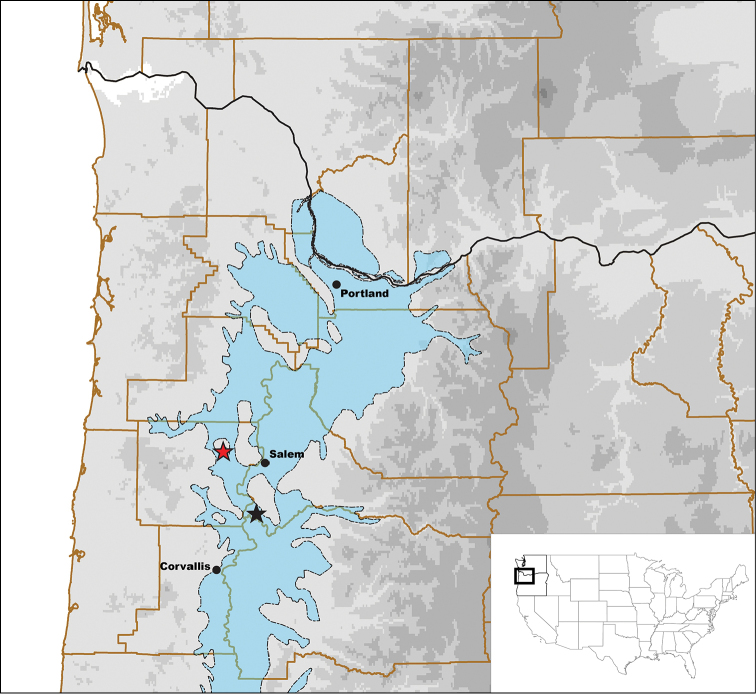
The two known collection localities of *Stygoporus oregonensis*. Oregon/Washington State boundary in black. County boundaries in brown. Blue shaded region outlined with a dotted line corresponds to Willamette Lowland basin-fill aquifers. Type locality indicated by red star with black border. New collection locality indicated by black star.

Two mostly intact specimens of *Stygoporus oregonensis* and fragments of additional individuals were recovered from accumulated sand and detritus in the filter of a residential well system (USA: Oregon: Marion County, Talbot, south of Talbot Road South). The well sits near an old oxbow of the Willamette River and the wellhead is located roughly 14 m below the surface. This site is roughly 27km SSE of the type locality (Fig. 2). Between 2014 and 2016, the accumulated material in the well filter was checked six times (Suppl. material 3). The first two surveys of the filtrate contained minute and pale beetle fragments assumed to be remnants of *Stygoporus oregonensis*. These fragments did not appear to contain any soft tissue; they may have died long before the filtrate was examined.

The mostly intact beetle specimens were both caught during the rainy winter months and contained soft tissue, which appeared to be suitable for DNA extraction and PCR sequencing. The specimens were found with the prothorax and head slightly separated from the rest of the body and the genitalia extruded as if they had expanded slightly. This damage may have occurred during depressurization: the removal of the filter causes a change in pressure from 8-10 psi to atmospheric pressure in approximately 2 seconds.

In addition to *Stygoporus oregonensis*, we recovered crustaceans (ostracods, copepods, and Bathynellacea), numerous oribatid mites, and a few other insects (Throscidae (Coleoptera), Chironomidae larvae (Diptera), and unattributed elytral fragments). While the Throscidae appears to be an obvious terrestrial contaminant, we could not determine if the other taxa are associated with the aquifer or not. The pair of pale elytra recovered in one of the samples (OSAC Lot 20160620-03) was markedly smaller and stouter than that of *Stygoporus oregonensis* and while it may have come from a surface dwelling species, it raises the possibility of additional undiscovered species inhabiting the aquifer.

### DNA extraction and sequencing

We extracted DNA from the two fairly intact specimens of *Stygoporus oregonensis* using DNeasy Blood and Tissue kits (Qiagen) following the manufacturer's protocols. Specimens were disarticulated between the abdomen and thorax prior to extraction; we did not grind any tissue, and thus the exoskeleton was preserved. We successfully amplified and sequenced six of the seven gene fragments used in Miller et al. (2013): 12S rRNA (12S), 16S rRNA (16S), cytochrome c oxidase I (COI), cytochrome c oxidase II (COII), *wingless*
(wg), and histone III (H3), but were unsuccessful at amplifying elongation factor 1-alpha. PCRs were performed in 25 microliter reactions on either an Eppendorf Mastercycler gradient or Mastercycler ProS using TaKaRa Ex Taq following manufacturer’s protocols. We used primer pairs and amplification conditions described in Miller et al. (2013) for 12S, 16S, COI (Pat/Jerry), COII, and H3, and Kanda et al. (2015) for *wg* and the barcoding region of COI (Suppl. material 4). PCR cleanup, quantification, and sequencing were performed at the University of Arizona’s Genomic and Technology Core Facility (UAGC) using a 3730 XL Applied Biosystems automatic sequencer.

### Sequence processing and phylogenetic analyses

Initial assembly of chromatograms was performed using Phred v. 0.020425.c (Green and Ewing 2002) and Phrap v. 0.990319 (Green 1999) as orchestrated by Mesquite v. 3.04 package Chromaseq v. 1.12 (Maddison and Maddison 2011, Maddison and Maddison 2015) with subsequent manual processing. *Stygoporus oregonensis* sequences were combined with single gene matrices from Miller et al. (2013). The taxon sampling used in Miller et al.’s (2013) study encompasses the morphological diversity of Hydroporinae, including numerous representatives of all currently recognized subtribes of Hydroporini (Suppl. material 5) and thus provides an excellent framework for inferring the phylogenetic placement of *Stygoporus oregonensis*.

12S and 16S matrices were aligned using MAFFT v. 7.130b (Katoh and Standley 2013) and the L-INS-i method. Alignment of protein-coding genes were performed manually since they either had no indels (COI, COII, and H3) or just a single inferred amino acid indel (*wg*). All nucleotide alignments were also combined into a single concatenated dataset.

Optimal data partition schemes and model of molecular evolution for protein-coding genes were inferred using PartitionFinder v. 1.1.1 (Lanfear et al. 2012) starting from an initial partition scheme based on codon position. Examined models were restricted to those available in RAxML, BIC was used to compare models, and the greedy algorithm was used for searches. Models for 12S and 16S were inferred using BIC implemented in jModelTest 2.0 (Darriba et al. 2012). PartitionFinder analysis was also conducted on the concatenated dataset starting with an initial partition scheme based on gene and codon. Optimal models and partitions for all datasets are presented in Table 1.

**Table 1.** Properties of phylogenetic datasets analyzed for this study. **NTaxa**: The number of taxa represented in the dataset. **Partitions**: Optimal partitioning scheme chosen by PartitionFinder**. NChar (BP)**: Number of characters (bases) in the aligned dataset/partition. **Model**: Optimal model of molecular evolution inferred by either jModelTest (12S, and 16S) or PartitionFinder (protein-coding genes).

Dataset	NTaxa	Partitions	NChar (BP)	Model
12S	49	*NA*	362	GTR+I+G
16S	50	*NA*	533	HKY+I+G
COI	44	(1) n1, n2	838	GTR+I+G
		(2) n3	418	GTR+G
COII	43	(1) n1, n2	450	GTR+I+G
		(2) n3	224	GTR+G
H3	50	(1) n1, n2, n3	328	GTR+I+G
*wg*	20	(1) n1, n2	306	GTR+I+G
		(2) n3	154	GTR+G
Concatenated	51	(1) 12S, 16S	895	GTR+I+G
		(2) n1 and n2 of all genes	1812	GTR+I+G
		(3) n3 of COI and COII	642	GTR+G

We conducted Maximum Likelihood (ML) analyses on single gene and concatenated datasets using RAxML v. 8.0.3 (Stamatakis 2014) implemented through the Mesquite package Zephyr v. 1.1 (Maddison and Maddison 2015) with optimal partition schemes and models of molecular evolution. When different models were chosen for different partitions, we applied the most complex model to the entire dataset. Since the HKY substitution model that was selected for 16S is not available in RAxML, we instead used GTR. We conducted 500 independent searches for the maximum likelihood tree and 1,000 bootstrap replicates on all datasets.

### Morphological methods

Methods for gross morphological examination and use of terms follow Miller (2005, 2016). The two extracted specimens were also used for morphological study of female internal reproductive characters. Female genitalia were dissected following DNA extraction, stained with 10% Chlorazol Black diluted in 75% ethanol, and examined on a slide in deionized water. During the course of study, the female genitalia of the recently acquired specimens were heavily damaged or lost accidentally after morphological features were recorded. Because of the extensive damage or loss, we chose not to image the genitalia. The female genital structures were mounted in Euparal on cardstock and pinned beneath the specimen.

The dorsal habitus image was taken with a Leica Z6 and JVC KY-F75U camera using Microvision’s Cartographer to take a stack of pictures at different focal planes. Stacking was performed using the PMax procedure implemented in Zerene Stacker (Zerene Systems). Removal of background and minor color adjustment was performed using Photoshop and Illustrator CS5 (Adobe).

### Data availability

All specimens examined in this study and the two DNA extractions are deposited in the Oregon State Arthropod Collection (OSAC), Oregon State University. Associated OSAC lot and voucher codes are given in Suppl. material 3. Final sequences for both specimens are available through GenBank (accession numbers KX882130-KX882141). Matrices used in the analyses are available as supplemental content (Suppl. material 6: MatricesForAnalyses.nex).

## Results

### Additional morphological characters for Stygoporus oregonensis

Morphological characters discussed below are based on the original description of *Stygoporus oregonensis* (Larson and LaBonte 1994) and material examined for the present study; the latter allowed us to examine previously unstudied characters of the proventriculus and female genitalia.

The proventriculus of *Stygoporus oregonensis* has a simple transverse tooth similar to that of *Hydroporus* Clairville with fields of papillae laterally. The female genitalia are of hydroporine-type (Miller 2001) with elongate ductwork. The external genitalia lack laterotergites, gonocoxosternites are broadly triangular and finely setose ventrally with an anteriorly rounded projection, gonocoxae are unfused, slender basally, broadening apically to a narrowly rounded apex, with numerous minute apical setae. Internally, the bursa is small and lacks a ring-like sclerite, the spermathecal duct is elongate and slender for most of its length, broadening before attaching to the small bulbous spermatheca, and the shorter fertilization duct is similarly slender and inserts ventrally on the vagina posterior to the common oviduct.

### Phylogenetic placement of *Stygoporus oregonensis*

**Figure 3.**
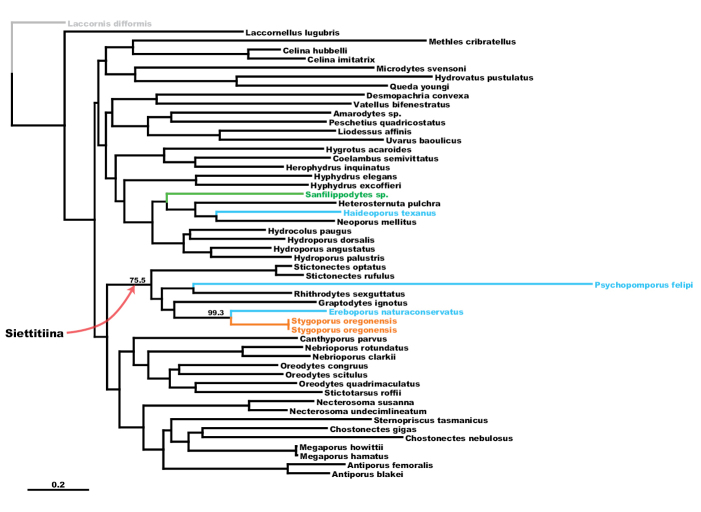
Maximum likelihood tree from concatenated dataset. Scale bar = 0.2 expected substitutions per position as estimated by RAxML. *Stygoporus oregonensis* in orange; other stygobitic dytiscids in blue; the epigean genus *Sanfilippodytes*, hypothesized by Larson and LaBonte (1994) to be the closest relative to *Stygoporus oregonensis*, in green. Bootstrap support given at nodes for Siettitiina and *Stygoporus oregonensis* + *Ereboporus naturaconservatus*.

**Figure 4.**
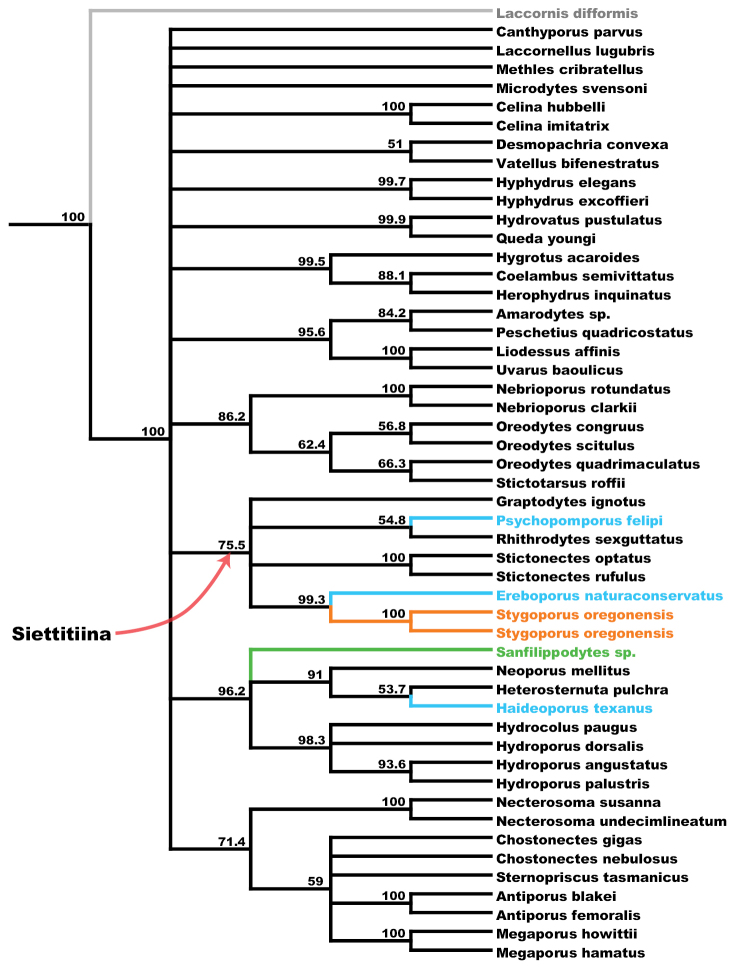
Majority rule consensus of 1,000 bootstrap replicates performed on concatenated dataset. Bootstrap percentages given for clades recovered with more than 50% support. Branches and taxa colored as in Figure 3.

The maximum likelihood (ML) tree of the concatenated dataset is shown in Figure 3 and majority rules consensus tree from 1000 bootstrap replicates is shown in Figure 4. ML trees and bootstrap consensus trees for single-gene datasets are provided in Suppl. material 1 and 2. ML
bootstrap support percentages (BSP) are summarized across phylogenetic reconstructions in Table 2 for hypotheses regarding the taxonomic placement of *Stygoporus oregonensis*.

**Table 2.** Bootstrap support for placement of *Stygoporus oregonensis*. Taxonomic hypotheses are in the first column. Bootstrap support given as a percentage for each hypothesis for all analyzed matrices. “Con” refers to the analysis of the concatenated matrix.

Taxonomic hypotheses	Con	12S	16S	COI	COII	H3	*wg*
*Stygoporus oregonensis* + *Ereboporus naturaconservatus*	99.3	79.2	41.1	60.1	70.7	86.0	90.0
*Stygoporus oregonensis + Sanfilippodytes*	0	0	0	0	0.2	3.0	0.5
Siettitiina including *Stygoporus oregonensis*	75.5	4.6	87.7	45.7	0	0	31.0
Siettitiina excluding *Stygoporus oregonensis*	0	0	1.3	1.2	0	0	0
*Stygoporus oregonensis* in Hydroporina	0	0.1	0	0	0	0	0

Maximum likelihood analysis of the concatenated dataset recovers *Stygoporus oregonensis* as sister to the Texas stygobite *Ereboporus naturaconservatus* (Figs 3, 4) with high bootstrap support (BSP=99.3). This clade is placed within the hydroporine subtribe Siettitiina, which is recovered with moderate support (BSP=75). Additional recovered genus or tribal-level groups largely correspond to the ML inference of phylogeny by Miller et al. (2013).

*Ereboporus naturaconservatus* and *Stygoporus oregonensis* are recovered as sister species in all single gene ML analyses (Suppl. material 1). This relationship is moderately to highly supported across single gene bootstrap analyses except in 16S (Table 2, Suppl. material 2). Although Siettitiina (*Graptodytes* group) is not equally well sampled for all genes, *Stygoporus oregonensis* and *Ereboporus naturaconservatus* are recovered within a monophyletic Siettitiina in ML analyses of 16S, COI, and *wg*. Support for Siettitiina (including *Stygoporus*) is high in bootstrap analyses of 16S but low to non-existent in other genes. *Stygoporus oregonensis* is never placed with *Sanfilippodytes* nor in Hydroporina in ML analyses of either the concatenated or single gene ML trees, and this hypothesis has no bootstrap support across analyses.

## Discussion

In their original description of *Stygoporus*, Larson and LaBonte (1994) placed it in the Hydroporini based on (1) posterior margin of metacoxal lobes continuous and sinuate, (2) posterior margin of metacoxal lobes unfused to abdominal ventrites II and III, (3) metafemur broadly separated from metacoxal lobe by large metatrochanter, (4) base of metafemur hidden ventrally by metacoxal lobe, and (5) male lateral lobes with a single segment. None of these morphological characters are synapomorphic for a tribal-level clade of Hydroporinae. Historically, Hydroporini included those Hydroporinae without a distinctive set of apomorphies, and clarifying relationships within Hydroporini has been a prominent goal of modern Dytiscidae systematics (Miller and Bergsten 2014). Recently, Miller and Bergsten (2014) reclassified the Hydroporini, giving genus group clades that were well supported with molecular and morphological data available higher-level names: Deronectina (*Deronectes* group), Hydroporina (*Hydroporus* group), Sternopriscina (*Necterosoma* group), and Siettitiina (*Graptodytes* group). While they did not have molecular sequence data for *Stygoporus*, they tentatively classified it within the Hydroporina (Miller and Bergsten 2014).

Larson and LaBonte (1994) hypothesized that *Stygoporus* is sister to *Sanfilippodytes* based on similar anteriorly produced metaventral processes, large metatrochanters, and habitat data. Contrary to this hypothesis, our molecular data places *Stygoporus oregonensis* within Siettitiina and not near Hydroporina and *Sanfilippodytes*. Though the phylogenetic analyses of Miller et al. (2013) and Miller and Bergsten (2014) strongly support the monophyly of Siettitiina, this clade is morphologically poorly defined. One potential synapomorphy is a ring-sclerite on the bursa copulatrix adjacent to the attachment of the spermathecal duct (Miller and Bergsten 2014). This structure is known to occur in *Ereboporus* and other siettitiines but is notably missing from *Graptodytes* Seidlitz (Miller et al. 2013), which is also the most diverse genus within the subtribe (Nilsson 2001). As in *Graptodytes*, the bursa copulatrix of *Stygoporus oregonensis* lacks a ring-like sclerite. We note that although the female genitalia in our specimen was damaged, it is clear that there is not a region along the bursa that looks more sclerotized or distinct from the remaining structure.

There are additional morphological characters in support of inclusion of *Stygoporus oregonensis* within Siettitiina, though it remains unclear whether these characters are strong synapomorphies for Siettitiina as a whole. In particular, the pronotum of *Stygoporus oregonensis* has prominent paralateral longitudinal creases or striae similar to many members of the larger group (e.g. *Graptodytes* Seidlitz, *Siettitia* Abeille de Perrin and *Etruscodytes* Mazza, Cianferoni, and Rocchi). The prosternal process of *Stygoporus oregonensis* contacts the anteriorly projecting and narrowly rounded metaventral process, resting dorsad to it and altogether looks remarkably similar to the Italian stygobite *Etruscodytes*. This region of the body has received much attention from biologists interested in stygobitic beetles (Miller et al. 2013; Spangler 1986), and these sclerites are intricately involved in locomotion, particularly wedging (Evans 1977). The similarity in form of these sclerites may be evidence of recent common ancestry, but this may also be the result of convergence as modifications to the ventral thoracic sclerites and the loss of a streamlined body are commonly observed patterns in distantly related subterranean diving beetles (Miller et al. 2009; Spangler 1986).

Other morphological features in *Stygoporus oregonensis* relevant to grouping within Hydroporini are known plesiomorphies. These are, for example, the simple transverse tooth of the proventriculus, the unfused, simple gonocoxae, the basally broad and apically narrowed elytral epipleuron, the male pro- and mesotarsomeres I-III with ventral adhesive setae, and the mesoventral fork separated from the anteromedial metaventral process. Most of these characters are unlike those observed in Deronectina and Sternopriscina, and the morphological evidence separating Hydroporina from Siettitiina is limited. Based on our observations, it appears that *Stygoporus* retains many plesiomorphies and placement based on morphological characters alone is difficult. However, the sequence data support the inclusion of *Stygoporus* within Siettitiina, and they decisively indicate that *Stygoporus* is closely related to *Ereboporus* among sampled species.

The Siettitiina has a predominantly Mediterranean and European distribution and includes many epigean species as well as other subterranean species (e.g. Ribera and Faille 2010). Intriguingly, the only presently known European stygobitic dytiscids are members of Siettitiina, including some species known only from wells and aquifers (Castro and Delgado 2001; Mazza et al. 2013; Ribera and Faille 2010). Aside from *Stygoporus oregonensis* and two described Texas subterranean aquifer endemics, Siettitiina are not represented in the New World, which suggests an ancient origin for these species (Miller et al. 2013). The mechanism and process behind this biogeographic pattern is not known. Conclusions invoking vicariance, dispersal, and extinction can certainly be applied to this pattern, but we prefer the practical hypothesis that at least part of this result is attributable to our ignorance. Instead of being dismayed, however, we are excited by the possibility that there are many unknown stygobitic beetles in aquifers between Oregon and Texas as well as other parts of the world for which little sampling of this habitat has been done.

## Supplementary materials

Supplementary material 1 Figure 1 Data type: Adobe PDF file Explanation note: Maximum likelihood trees for single gene datasets. Scale bar indicates the expected substitutions per site as estimated by RAxML. This dataset is made available under the Open Database License (http://opendatacommons.org/licenses/odbl/1.0/). The Open Database License (ODbL) is a license agreement intended to allow users to freely share, modify, and use this Dataset while maintaining this same freedom for others, provided that the original source and author(s) are credited.Kojun Kanda, R. Antonio Gomez, Richard Van Driesche, Kelly B. Miller, David R. Maddison

Supplementary material 2 Figure 2 Data type: Adobe PDF file Explanation note: Majority rule consensus of 1,000 bootstrap replicates performed on single gene datasets. Bootstrap percentage given for clades recovered with more than 50% support. This dataset is made available under the Open Database License (http://opendatacommons.org/licenses/odbl/1.0/). The Open Database License (ODbL) is a license agreement intended to allow users to freely share, modify, and use this Dataset while maintaining this same freedom for others, provided that the original source and author(s) are credited.Kojun Kanda, R. Antonio Gomez, Richard Van Driesche, Kelly B. Miller, David R. Maddison

Supplementary material 3 Table 1 Data type: MS Word file Explanation note: Collection and specimen data for material examined in this study. This dataset is made available under the Open Database License (http://opendatacommons.org/licenses/odbl/1.0/). The Open Database License (ODbL) is a license agreement intended to allow users to freely share, modify, and use this Dataset while maintaining this same freedom for others, provided that the original source and author(s) are credited.Kojun Kanda, R. Antonio Gomez, Richard Van Driesche, Kelly B. Miller, David R. Maddison

Supplementary material 4 Table 2 Data type: MS Word file Explanation note: PCR primers and amplification conditions for sampled gene fragments. This dataset is made available under the Open Database License (http://opendatacommons.org/licenses/odbl/1.0/). The Open Database License (ODbL) is a license agreement intended to allow users to freely share, modify, and use this Dataset while maintaining this same freedom for others, provided that the original source and author(s) are credited.Kojun Kanda, R. Antonio Gomez, Richard Van Driesche, Kelly B. Miller, David R. Maddison

Supplementary material 5 Table 3 Data type: MS Word file Explanation note: Taxa from Miller et al. (2013) sampled in this study with updated tribal and subtribal classification of Miller and Bergsten (2014). This dataset is made available under the Open Database License (http://opendatacommons.org/licenses/odbl/1.0/). The Open Database License (ODbL) is a license agreement intended to allow users to freely share, modify, and use this Dataset while maintaining this same freedom for others, provided that the original source and author(s) are credited.Kojun Kanda, R. Antonio Gomez, Richard Van Driesche, Kelly B. Miller, David R. Maddison

Supplementary material 6 Data availability Data type: NEXUS file Explanation note: NEXUS formatted single-gene and concatenated nucleotide sequence alignments. This dataset is made available under the Open Database License (http://opendatacommons.org/licenses/odbl/1.0/). The Open Database License (ODbL) is a license agreement intended to allow users to freely share, modify, and use this Dataset while maintaining this same freedom for others, provided that the original source and author(s) are credited.Kojun Kanda, R. Antonio Gomez, Richard Van Driesche, Kelly B. Miller, David R. Maddison
