# Supplementary figures and images for: Phylogenetic placement of the Pacific Northwest subterranean endemic diving beetle Stygoporus oregonensis Larson & LaBonte (Dytiscidae, Hydroporinae)
Source: Zookeys. 2016 Nov 16;(632):75–91. doi: 10.3897/zookeys.632.9866 (PMC5126547; doi:10.3897/zookeys.632.9866)

# 12S

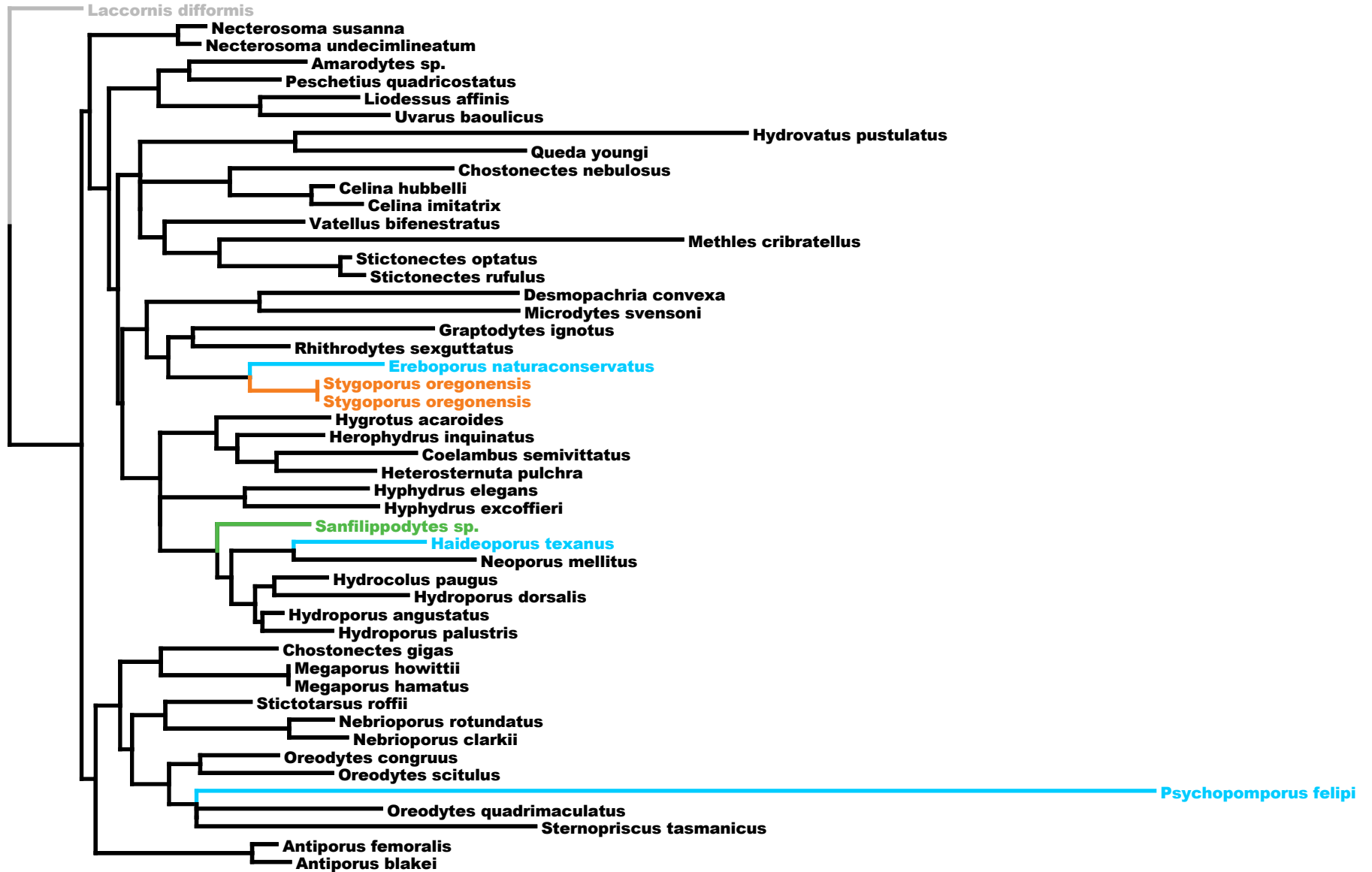

0.1

16S

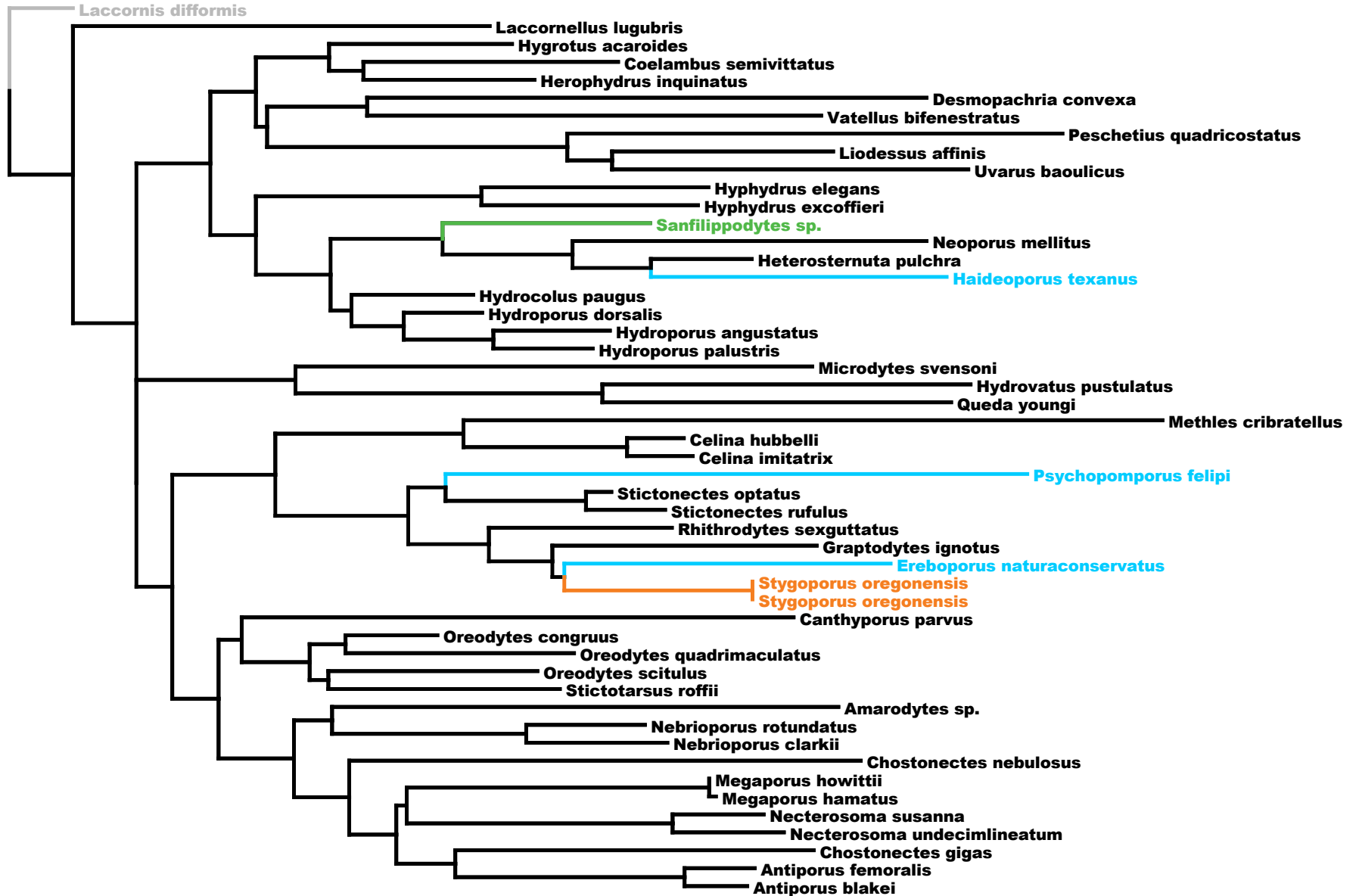

0.05

# COI

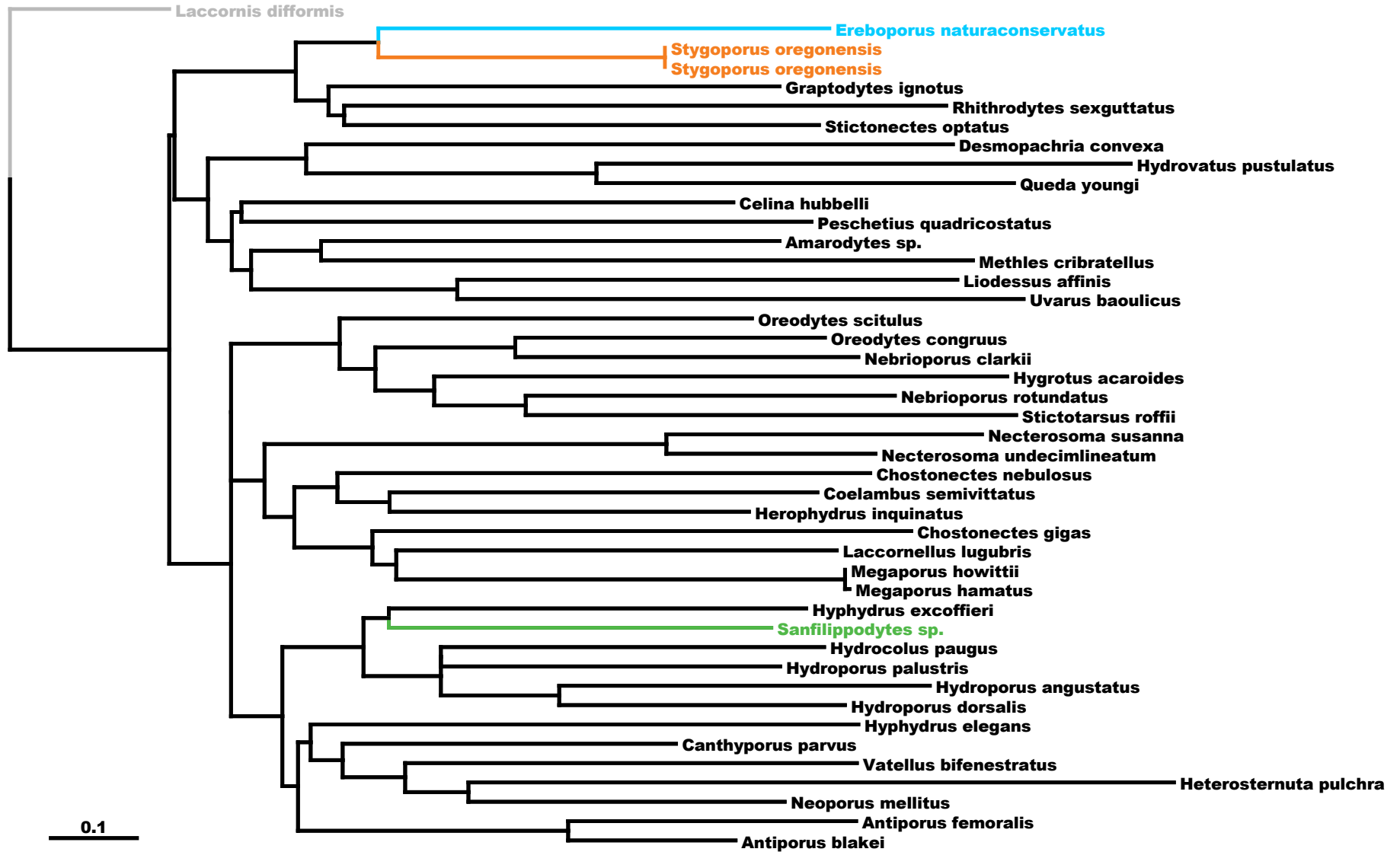

COII

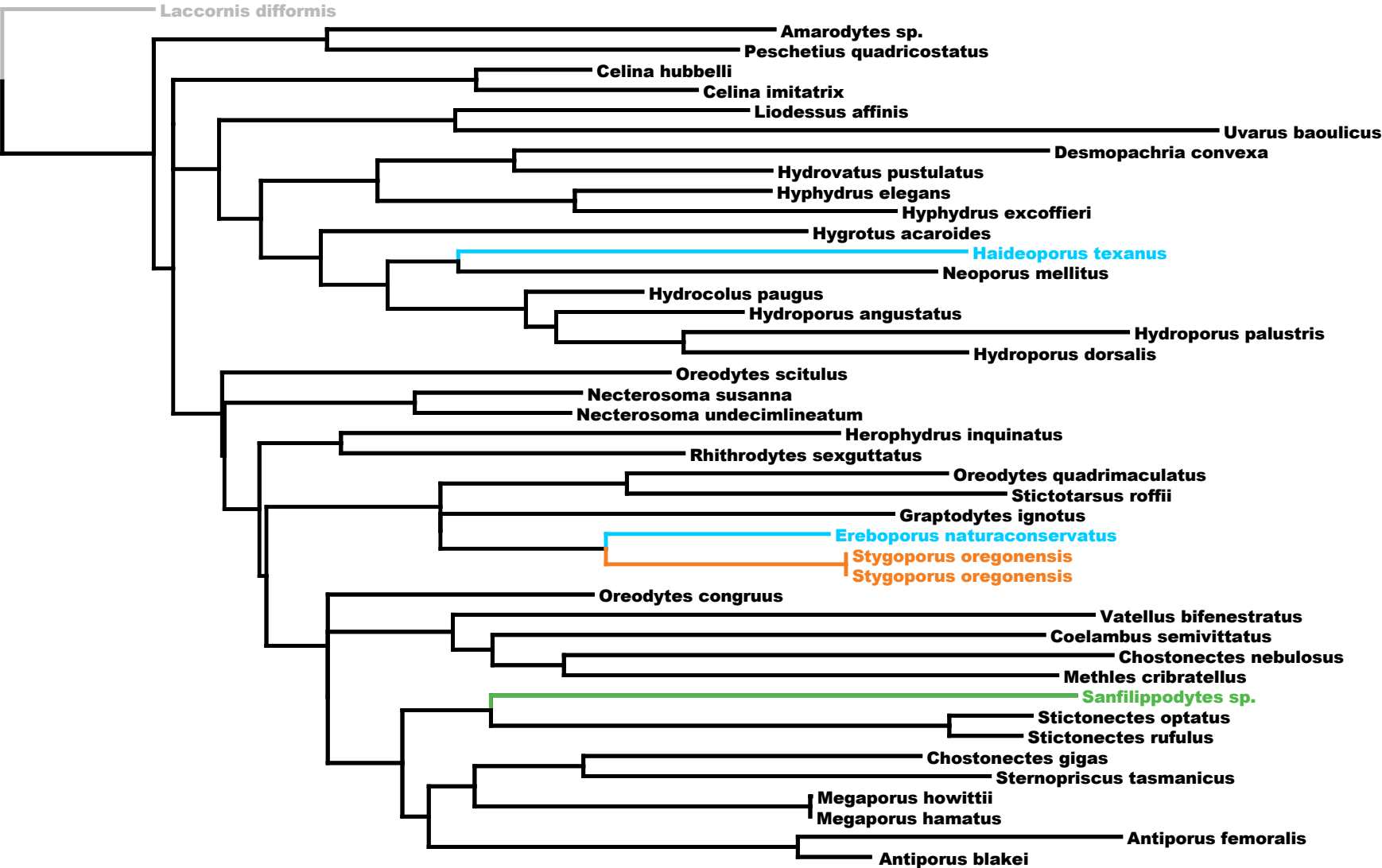

1.0

# H3

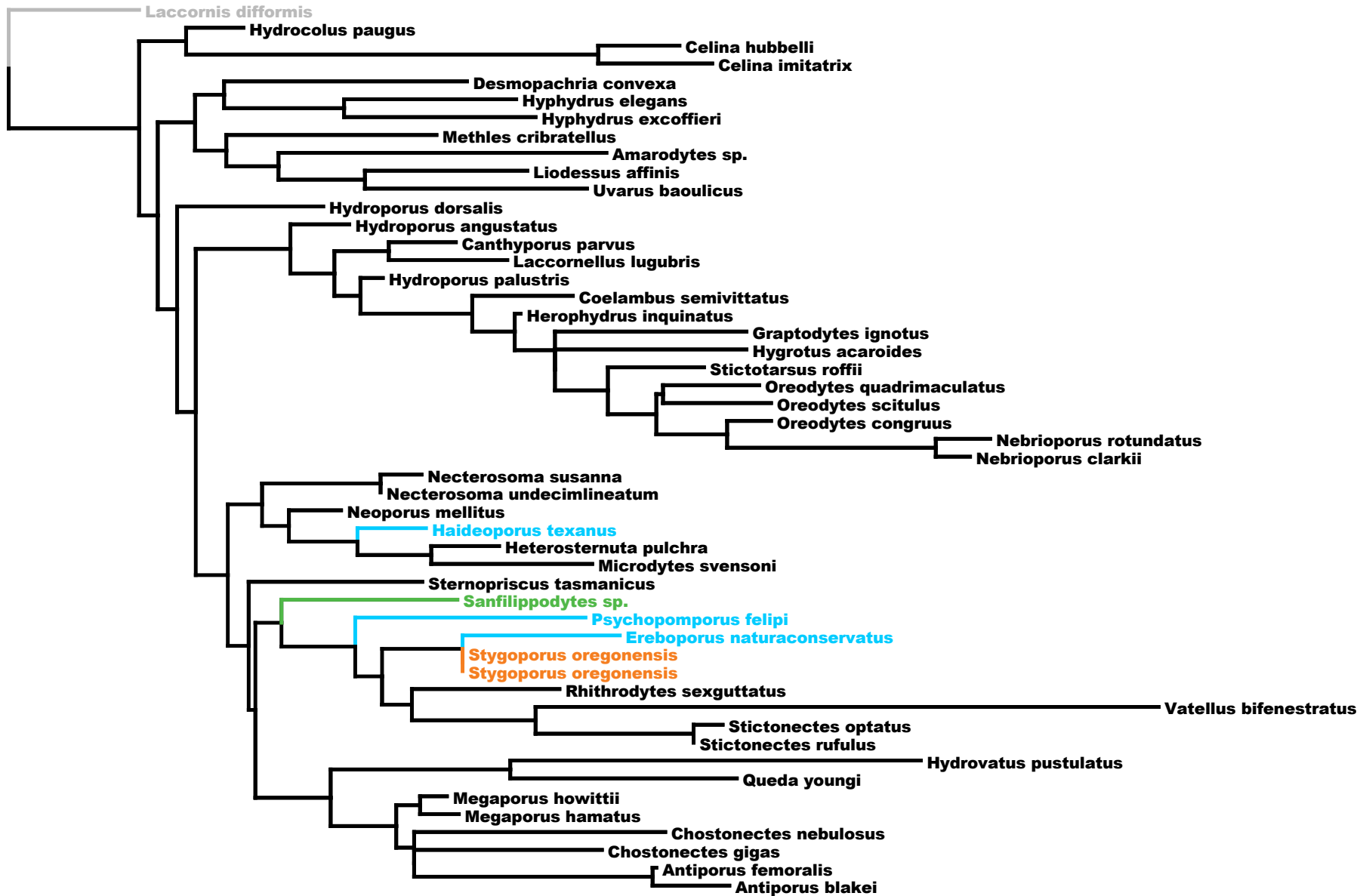

0.1

**wg**

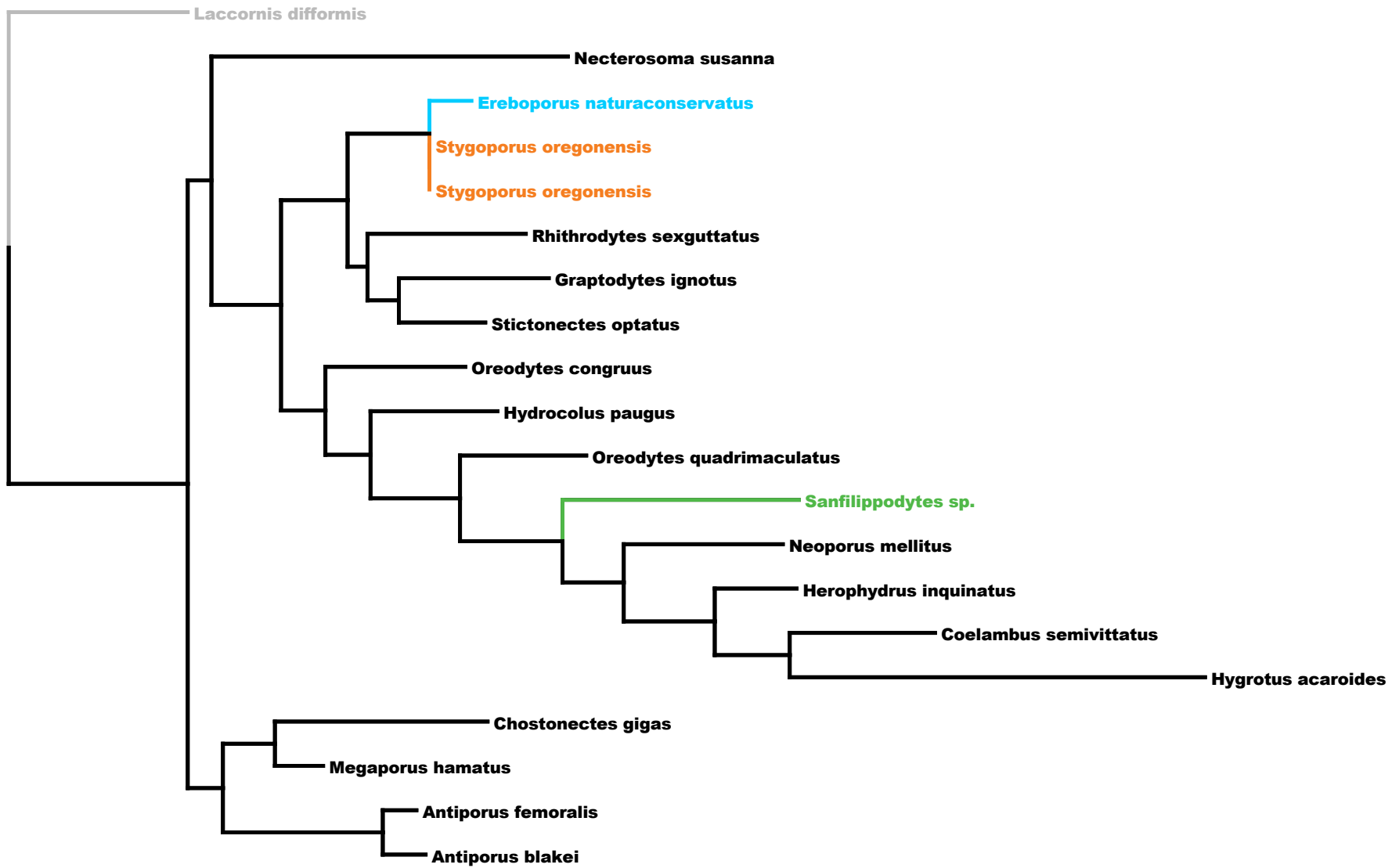

Supplement: Supplementary material 1 — Figure 1 [file zookeys-632-075-s001.pdf]

12S

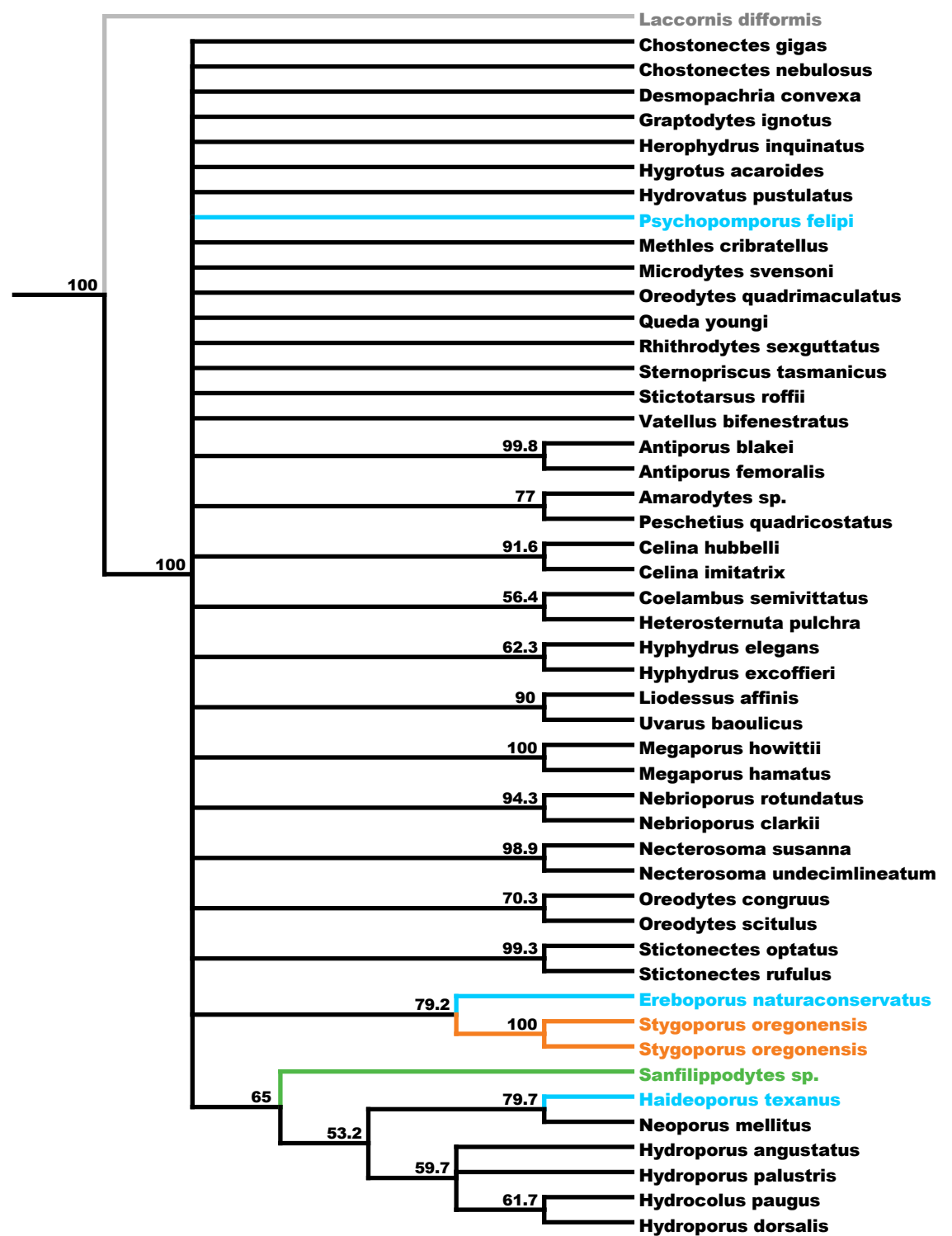

16S

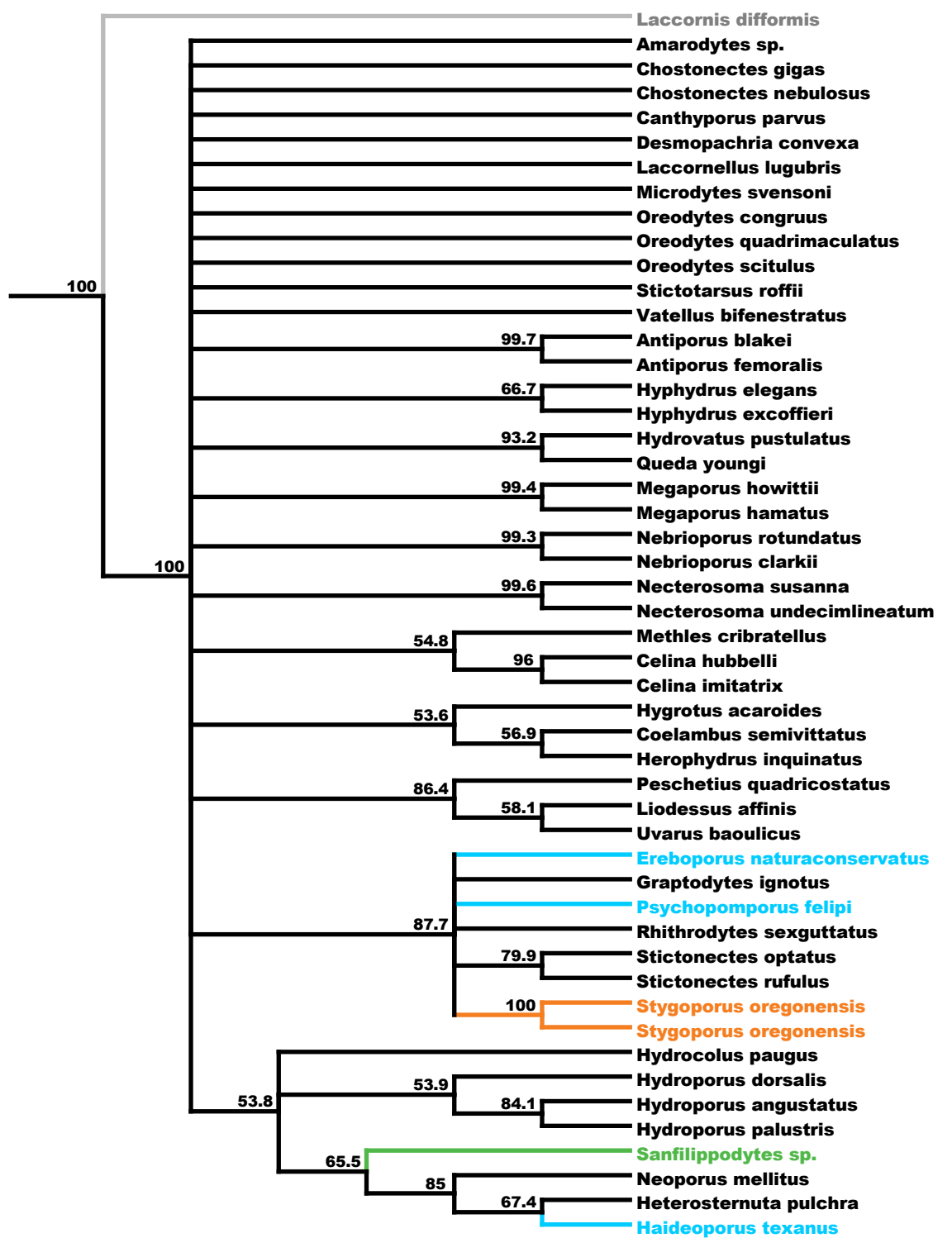

COI

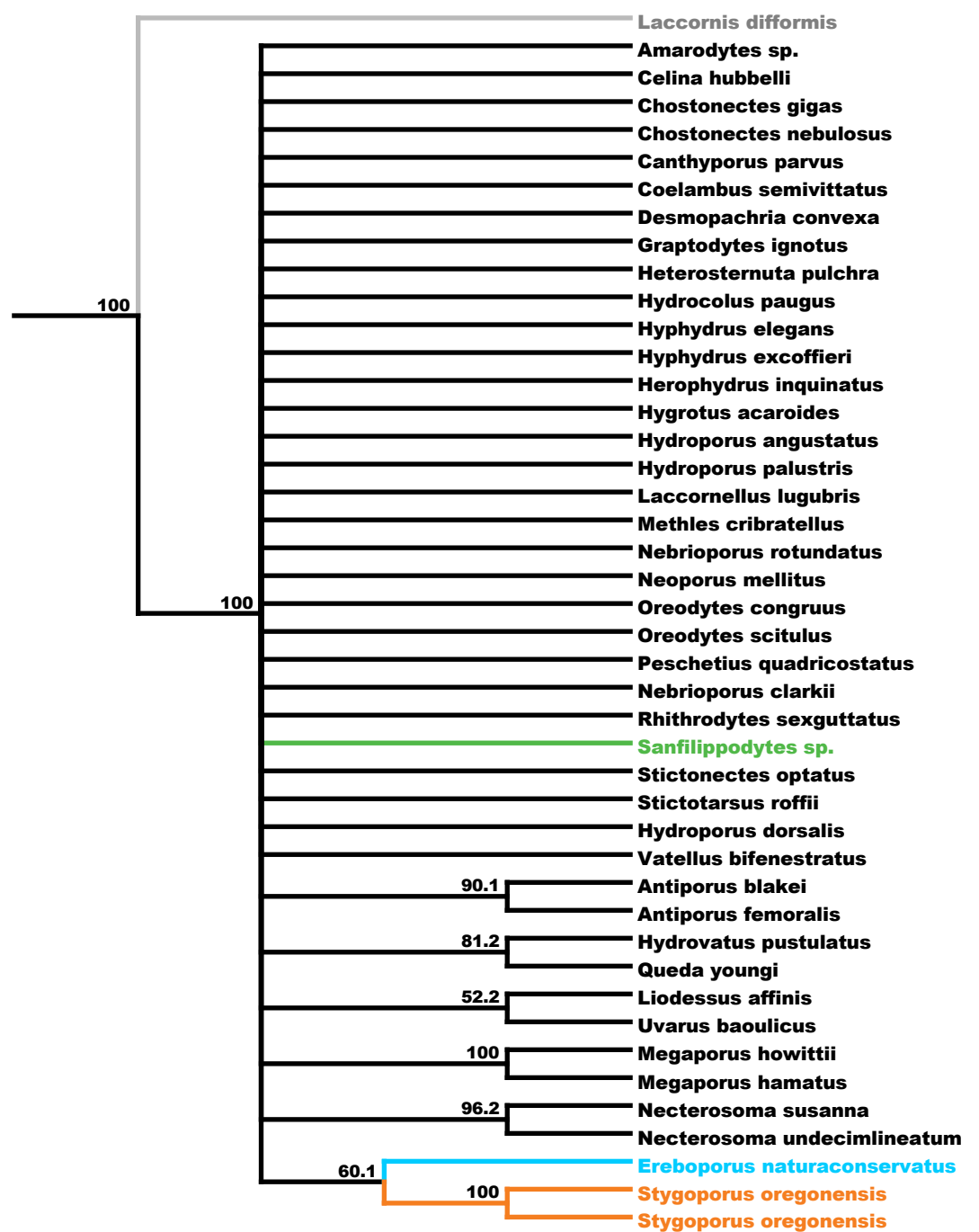

COII

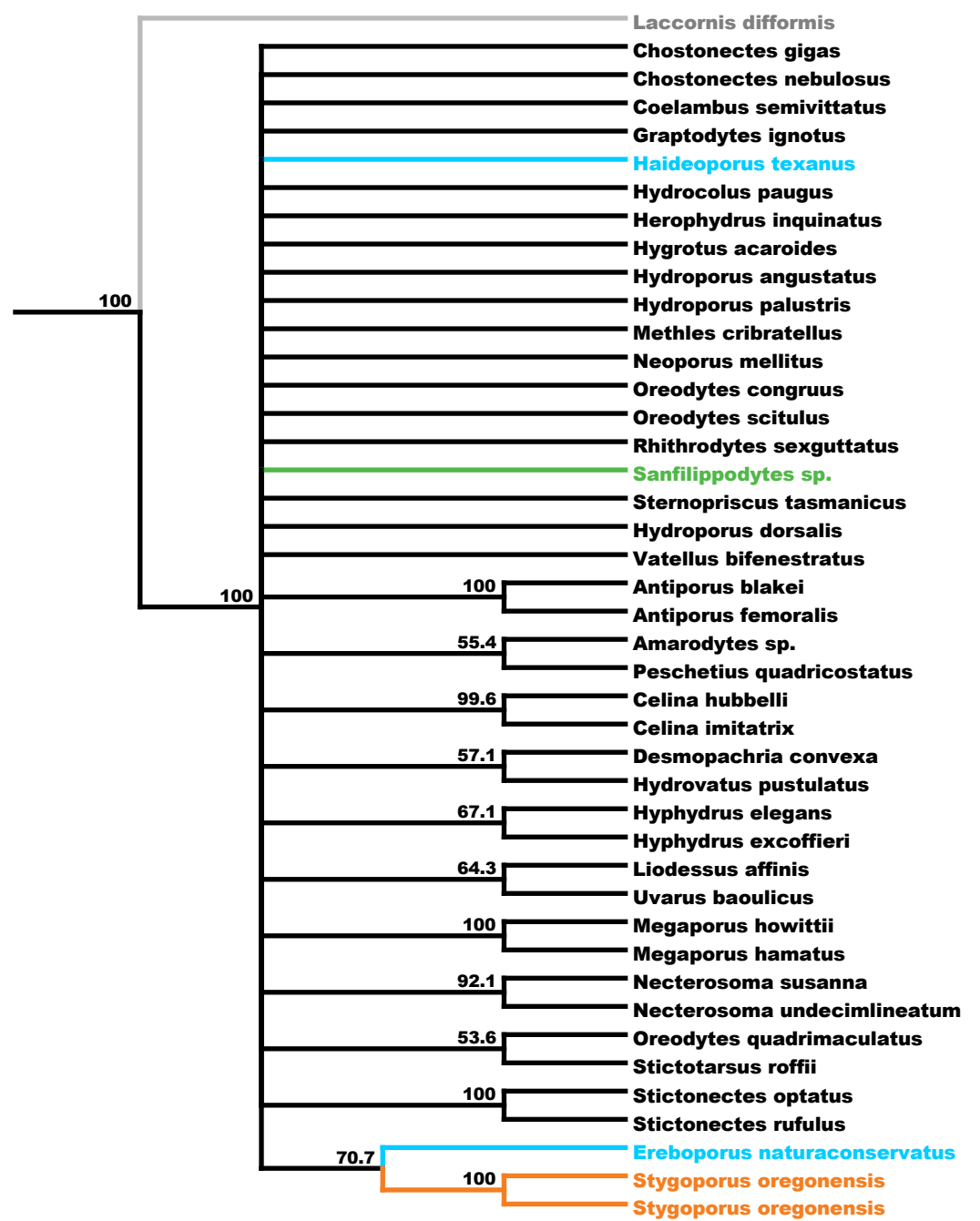

H3

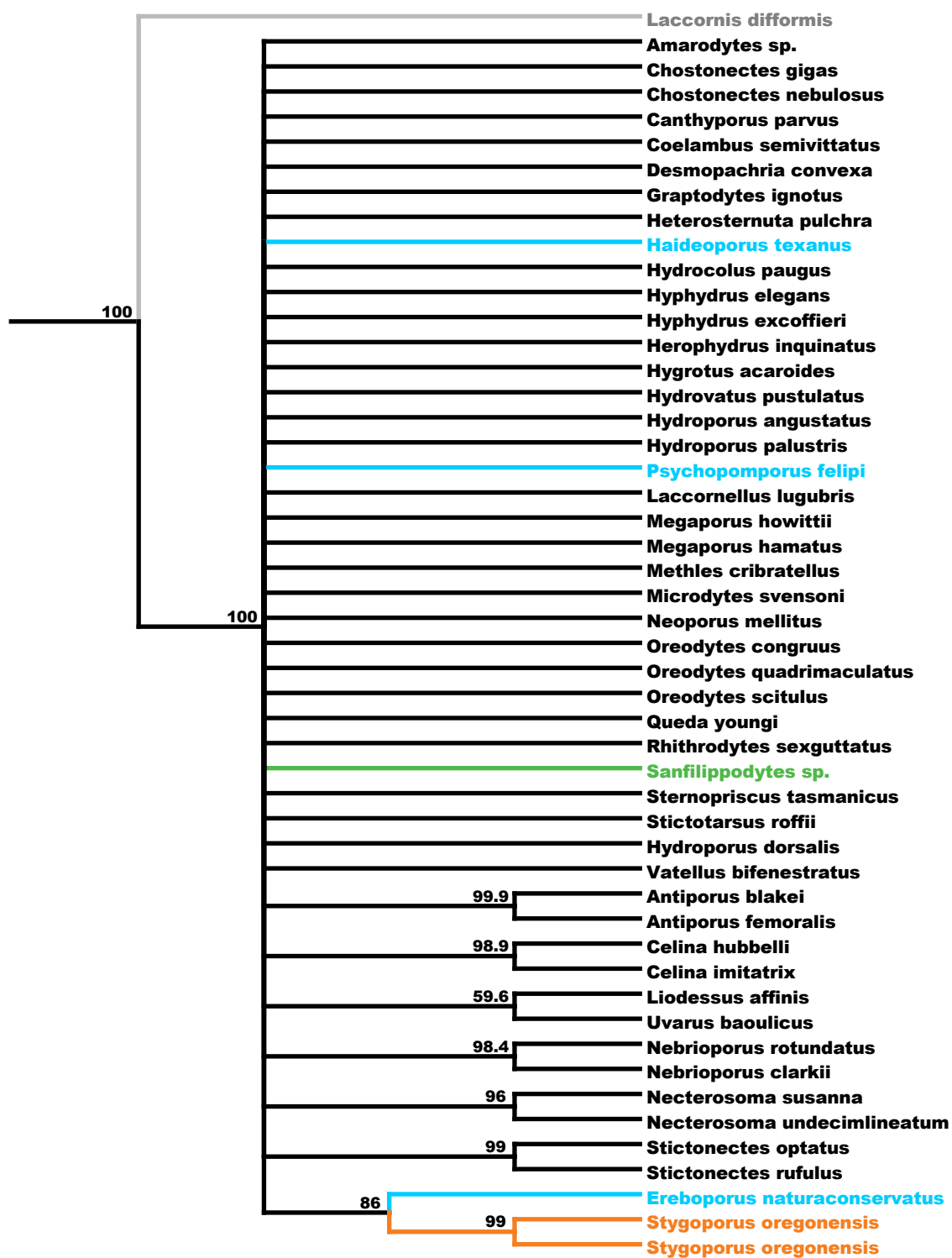

**wg**

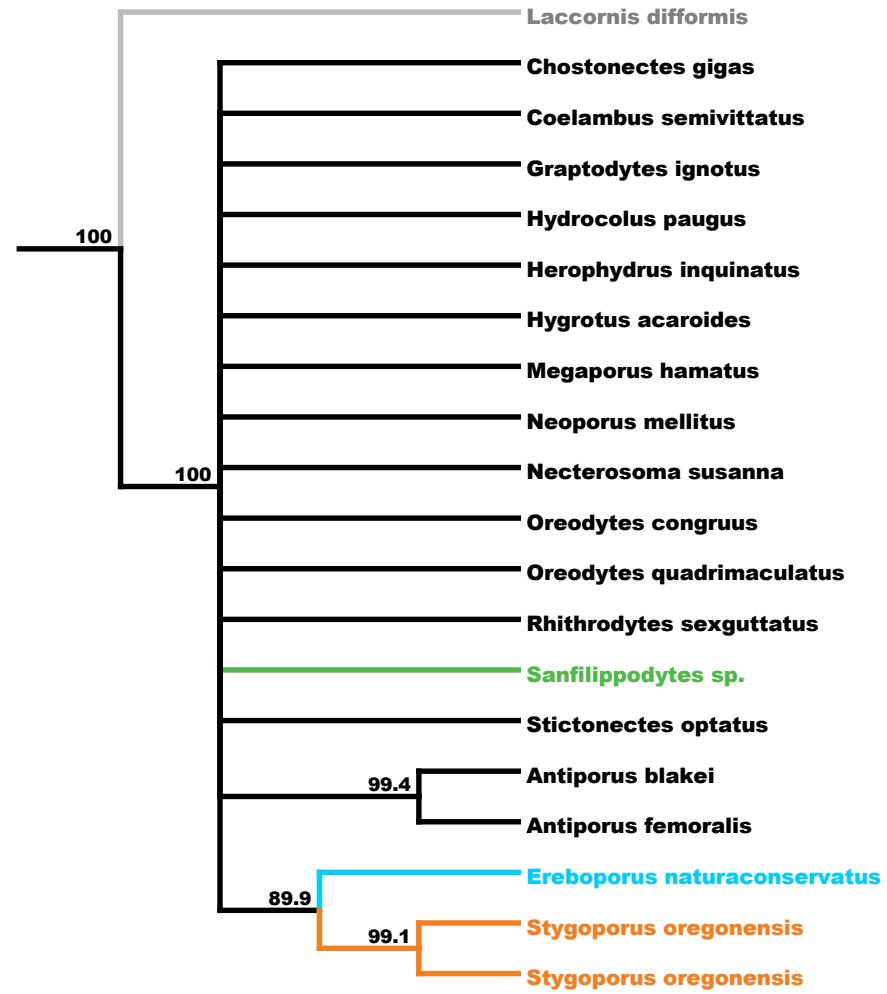

Supplement: Supplementary material 2 — Figure 2 [file zookeys-632-075-s002.pdf]
