# Supplementary material for: Phylogenetic placement of the Pacific Northwest subterranean endemic diving beetle Stygoporus oregonensis Larson & LaBonte (Dytiscidae, Hydroporinae)
Source: Zookeys. 2016 Nov 16;(632):75–91. doi: 10.3897/zookeys.632.9866 (PMC5126547; doi:10.3897/zookeys.632.9866)
Supplement: Supplementary material 3 — Table 1 [file zookeys-632-075-s003.docx]

**Supplemental Table 1.** Collection and specimen data for material examined in this study. All specimens were collected from the same wellhead (USA: Oregon: Marion County, Talbot, south of Talbot Road South) but on different dates. **Collection Date**: the date the debris in the filter was removed.  **OSAC Lot/Specimen Code**: Lot and voucher codes for material deposited in the Oregon State Arthropod Collection (Oregon State University, Corvallis OR.). DNA voucher codes used by KK and DRM given in parentheses. **Taxa Recovered**: Taxa that were recovered from the debris in the well filter and deposited in OSAC. **Specimen Notes**: Condition of specimens when they were collected and how they are stored. **Associated Data**: Published data associated with material from the lot or specimen.

| **Collection Date** | **OSAC Lot/Specimen Code** | **Taxa Recovered** | **Specimen Notes** | **Associated Data** |
| --- | --- | --- | --- | --- |
| 13.iv.2014 | OSAC Lot 20160620-01 | *S. oregonensis* | Fragments from three specimens stored in 75% EtOH. |  |
| 19.x.2014 | OSAC Lot 20160620-02, OSAC_000974170 | *S. oregonensis*, oribatid acari, copepod | Fragments of one *S. oregonensis,* which were subsequently card mounted (OSAC_000974170). Specimens of other taxa stored in 75% EtOH. |  |
| 27.xii.2014 | OSAC Lot 20160620-03, OSAC_000974168 (KKDNA0345) | *S. oregonensis, oribatid acari, copepod, Coleoptera* | One intact *S. oregonensis* contained soft tissue (OSAC_000974168). DNA was extracted from the specimen and it is now cardmounted with abodmen and genitalia stored in a gel capsule. Specimens of additional taxa stored in 75% EtOH. In this sample is a pair of pale elytra, which cannot be attributed to any specific family. | OSAC_000974168 imaged for Fig. 1 Genbank accessions: KX882130, KX882132, KX882134, KX882136, KK882138, KX882140 |
| 25.vii.2015 | OSAC Lot 20160620-04 | *S. oregonensis,* oribatid acari, Bathynellacea | Thorax and abdomen of one *S. oregonensis*. All specimens stored in 75% EtOH. |  |
| 12.xi.2015 | OSAC_000974169 (DNA4820) | *S. oregonensis* | One intact *S. oregonensis* with soft tissue was recovered. DNA was extracted from the specimen. Disarticulated cuticle stored in 75% EtOH. | Genbank accessions: KX882131, KX882133, KX882135,  KX882137,  KX882139  KX882141 |
| 15.vi.2016 | OSAC Lot 20160620-05 | Oribatid acari, Throscidae (Coleoptera), Chironomidae (Diptera) larvae, copepod, Bathynellacea | The throscid is most likely a contaminant from above ground. All specimens stored in 75% EtOH. |  |
