# Supplementary material for: Phylogenetic placement of the Pacific Northwest subterranean endemic diving beetle Stygoporus oregonensis Larson & LaBonte (Dytiscidae, Hydroporinae)
Source: Zookeys. 2016 Nov 16;(632):75–91. doi: 10.3897/zookeys.632.9866 (PMC5126547; doi:10.3897/zookeys.632.9866)
Supplement: Supplementary material 4 — Table 2 [file zookeys-632-075-s004.docx]

**Supplemental Table 2.** PCR primers and amplification conditions for sampled gene fragments. **Gene**: gene name of sequenced fragments. **Primer**: published name of primer. **Dir.**: whether primer is a forward (F) or reverser (R) primer. **Cit.**: source of primers. Number in brackets refer to the literature listed below the table. **Primer Sequence (5’-3’)**: nucleotide sequence of the primer**. Amplification Condition**: amplification strategy for the gene fragment. All reactions begin with 2-minute denaturation at 94˚ and end with a 5-minute extension at 72˚C. 38 cycles were performed in each reaction. All cycles begin with a 20 second denaturation at 94˚C, followed by 20 seconds of primer annealing with annealing temperature (AT) varying between primer pairs, ending with extension at 72˚C with extension time (ET) varying between primer pairs.

| **Gene** | **Primer** | **Dir.** | **Cit.** | **Primer Sequence (5’-3’)** | **Amplification Condition** |
| --- | --- | --- | --- | --- | --- |
| 12S | 12Sai | F | [1] | AAACTACGATTAGATACCCTATTAT | AT=52˚C, ET=60 sec. |
|  | 12Sbi | R | [1] | AAGAGCGACGGGCGATGTGT |  |
| 16S | 16S A | F | [1] | CGCCTGTTTATCAAAAACA | AT=52˚C, ET=60 sec. |
|  | 16S B | R | [1] | CTCCGGTTTGAACTCAGACA |  |
| COI | C1-J-2183 (“Jerry”) | F | [2] | CAACATTTATTTTGATTTTTTGG | Amplify and sequence two fragments.  Frag. 1: use Jerry/Pat, AT=47˚C, ET=60 sec. Frag. 2 (Barcoding region of COI): use LCO1490/HCO2198, AT=52˚C, ET=60 sec. |
|  | TL2-N-3014 (“Pat”) | R | [2] | TCCAATGCACTAATCTGCCATATTA |  |
|  | LCO1490 | F | [3] | GGTCAACAAATCATAAAGATATTGG |  |
|  | HCO2198 | R | [3] | TAAACTTCAGGGTGACCAAAAAATCA |  |
| COII | F-lue | F | [4] | TCTAATATGGCAGATTAGTGC | AT=52˚C, ET=60 sec. |
|  | R-lys | R | [4] | GAGACCAGTACTTGCTTTCAGTCATC |  |
| H3 | Haf | F | [5] | ATGGCTCGTACCAAGCAGACGGC | AT=50˚C, ET=45 sec. |
|  | Har | R | [5] | ATATCCTTGGGCATGATGGTGAC |  |
| wg | wg550F | F | [6] | ATGCGTCAGGARTGYAARTGYCAYGGYATGTC | Nested PCR. Use wg550F/wgABR, AT=54˚C, ET=60 sec. Use PCR product from first reaction as template for a second PCR with wg578F/wgABRZ, AT=54˚C, ET=45 sec. |
|  | wgABR | R | [7] | YTCGCAGCACCARTGGAA |  |
|  | wg578F | F | [7] | TGCACNGTGAARACYTGCTGGATG |  |
|  | wgABRZ | R | [6] | CACTTNACYTCRCARCACCARTG |  |

[1] Svenson GJ, Whiting MF (2004) Phylogeny of Mantodea based on molecular data: evolution of a charismatic predator. Systematic Entomology 29: 359-370.

[2] Simon C, Frati F, Beckenbach A, Crespi B, Liu H, Flook P (1994) Evolution, weighting, and phylogenetic utility of mitochondrial gene sequences and a compilation of conserved polymerase chain reaction primers. Annals of the entomological Society of America 87: 651-701.

[3] Hebert PD, Cywinska A, Ball SL (2003) Biological identifications through DNA barcodes. Proceedings of the Royal Society of London B: Biological Sciences 270: 313-321.

[4] Whiting MF (2002) Mecoptera is paraphyletic: multiple genes and phylogeny of Mecoptera and Siphonaptera. Zoologica Scripta 31: 93-104.

[5] Colgan D, McLauchlan A, Wilson G, Livingston S, Edgecombe G, Macaranas J, Cassis G, Gray M (1998) Histone H3 and U2 snRNA DNA sequences and arthropod molecular evolution. Australian Journal of Zoology 46: 419-437.

[6] Wild AL, Maddison DR (2008) Evaluating nuclear protein-coding genes for phylogenetic utility in beetles. Molecular Phylogenetics and Evolution 48: 877-891.

[7] Ward PS, Downie DA (2005) The ant subfamily Pseudomyrmecinae (Hymenoptera: Formicidae): phylogeny and evolution of big‐eyed arboreal ants. Systematic Entomology 30: 310-335.
