# Supplementary material for: Phylogenetic placement of the Pacific Northwest subterranean endemic diving beetle Stygoporus oregonensis Larson & LaBonte (Dytiscidae, Hydroporinae)
Source: Zookeys. 2016 Nov 16;(632):75–91. doi: 10.3897/zookeys.632.9866 (PMC5126547; doi:10.3897/zookeys.632.9866)
Supplement: Supplementary material 5 — Table 3 [file zookeys-632-075-s005.docx]

**Supplemental Table 3.** Taxa from Miller et al. (2013) sampled in this study with updated tribal and subtribal classification of Miller and Bergsten (2014).

| **Tribe** | **Subtribe** | **Taxa** |
| --- | --- | --- |
| Bidessini |  | *Amarodytes sp.* |
| Bidessini |  | *Liodessus affinis* |
| Bidessini |  | *Peschetius quadricostatus* |
| Bidessini |  | *Uvarus baoulicus* |
| Hydroporini | Deronectina | *Nebrioporus clarkii* |
| Hydroporini | Deronectina | *Nebrioporus rotundatus* |
| Hydroporini | Deronectina | *Oreodytes congruus* |
| Hydroporini | Deronectina | *Oreodytes quadrimaculatus* |
| Hydroporini | Deronectina | *Oreodytes scitulus* |
| Hydroporini | Deronectina | *Stictotarsus roffii* |
| Hydroporini | Hydroporina | *Haideoporus texanus* |
| Hydroporini | Hydroporina | *Heterosternuta pulcher* |
| Hydroporini | Hydroporina | *Hydrocolus paugus* |
| Hydroporini | Hydroporina | *Hydroporus angustatus* |
| Hydroporini | Hydroporina | *Hydroporus dorsalis* ******* |
| Hydroporini | Hydroporina | *Hydroporus palustris* |
| Hydroporini | Hydroporina | *Neoporus mellitus* |
| Hydroporini | Hydroporina | *Sanfilippodytes sp.* |
| Hydroporini | Siettitiina | *Ereboporus naturaconservatus* |
| Hydroporini | Siettitiina | *Graptodytes ignotus* |
| Hydroporini | Siettitiina | *Psychopomporus felipi* |
| Hydroporini | Siettitiina | *Rhithrodytes sexguttatus* |
| Hydroporini | Siettitiina | *Stictonectes optatus* |
| Hydroporini | Siettitiina | *Stictonectes rufulus* |
| Hydroporini | Sternopriscina | *Antiporus blakei* |
| Hydroporini | Sternopriscina | *Antiporus femoralis* |
| Hydroporini | Sternopriscina | *Chostonectes gigas* |
| Hydroporini | Sternopriscina | *Chostonectes nebulosus* |
| Hydroporini | Sternopriscina | *Megaporus hamatus* |
| Hydroporini | Sternopriscina | *Megaporus howittii* |
| Hydroporini | Sternopriscina | *Necterosoma susanna* |
| Hydroporini | Sternopriscina | *Necterosoma undecimlineatum* |
| Hydroporini | Sternopriscina | *Sternopriscus tasmanicus* |
| Hydrovatini |  | *Hydrovatus pustulatus* |
| Hydrovatini |  | *Queda youngi* |
| Hygrotini |  | *Coelambus semivittatus* |
| Hygrotini |  | *Herophydrus inquinatus* |
| Hygrotini |  | *Hygrotus acaroides* |
| Hyphydrini |  | *Desmopachria convexa* |
| Hyphydrini |  | *Hyphydrus elegans* |
| Hyphydrini |  | *Hyphydrus excoffieri* |
| Hyphydrini |  | *Microdytes svensoni* |
| Laccornellini |  | *Canthyporus parvus* |
| Laccornellini |  | *Laccornellus lugubris* |
| Laccornini |  | *Laccornis difformis* |
| Methlini |  | *Celina hubbelli* |
| Methlini |  | *Celina imitatrix* |
| Methlini |  | *Methles cribratellus* |
| Vatellini |  | *Vatellus bifenestratus* |

* Miller et al. (2013) use *Suphrodytes dorsalis*. This has since been synonymized with *Hydroporus*.
